# Long COVID Among Undocumented Latino Immigrant Populations in the Emergency Department

**DOI:** 10.1001/jamanetworkopen.2024.38806

**Published:** 2024-10-11

**Authors:** Karen P. Reyes, Zubaid Rafique, Brian Chinnock, Vijaya Arun Kumar, Michael Gottlieb, Kristin L. Rising, Robert M. Rodriguez

**Affiliations:** 1 School of Medicine, University of California, San Francisco; 2 Henry JN Taub Department of Emergency Medicine, Baylor College of Medicine, Houston, Texas; 3 Department of Emergency Medicine, University of California, San Francisco, Fresno; 4 Department of Emergency, Wayne State University School of Medicine, Detroit, Michigan; 5 Department of Emergency Medicine, Rush University Medical Center, Chicago, Illinois; 6 Department of Emergency Medicine, Thomas Jefferson University, Philadelphia, Pennsylvania; 7 Department of Emergency Medicine, University of California, San Francisco

## Abstract

This cross-sectional study assesses the understanding of and access to care for long COVID symptoms among undocumented Latino immigrants in US emergency departments (EDs).

## Introduction

Although many investigators have examined post–COVID-19 condition (long COVID) and its effects in the general population, to our knowledge, no studies have investigated its effect on one of the largest underserved US populations: undocumented Latino immigrants.^1^ This group comprises 7% of the US population and has high rates of uninsurance, limited access to primary care, and language barriers when seeking health care.^2,3,4^ Anti-immigrant political rhetoric and immigrants’ fear of discovery of their undocumented status when accessing health care make evaluation of this population more challenging.^5^ We leveraged emergency departments (EDs) as the primary health care access point for underserved populations to address this gap.^2^

## Methods

This cross-sectional study using a verbally administered survey in English and Spanish was conducted at 9 EDs in 7 US cities from May to December 2023. The institutional review board at each site approved the study. Oral informed consent was obtained. We followed the STROBE reporting guideline.

During 8-hour time blocks, staff screened non–critically ill ED patients aged 18 years or older and enrolled those who had a self-reported positive COVID-19 test result 1 month or more before their visit (eMethods and eAppendices 1 and 2 in Supplement 1 give exclusions and the survey instrument). We categorized participants into 3 groups (undocumented Latino immigrants, Latino legal residents and citizens [LRCs], and non-Latino LRCs) using the questions, “Are you a US citizen?” and “Do you identify as Latino/a?”

Key outcomes were patients’ knowledge and understanding of the term *long COVID*; prevalence and impact of long COVID symptoms, defined as symptoms lasting over 1 month after acute infection (per the CDC definition at the initiation of this study)^6^; and patients’ prior receipt of care for long COVID symptoms. We reported findings as proportions and compared differences in proportions with 95% CIs. Analyses were performed using Python, version 3.8.5.

## Results

Of 844 eligible patients, 818 (97.0%) agreed to participate. Of these, 136 (16.6%) were undocumented Latino immigrants; 160 (19.6%), Latino LRCs; and 522 (63.8%), non-Latino LRCs. Undocumented Latino immigrants were more commonly uninsured (35 [25.7%] vs 13 [8.1%] and 21 [4.0%]) and lacking primary care (39 [28.7%] vs 32 [20.1%] and 50 [9.6%]) compared with Latino and non-Latino LRCs (Table 1). Compared with non-Latino LRCs, undocumented Latino immigrants and Latino LRCs were less likely to have knowledge and understanding of long COVID (182 of 518 [35.1%] vs 15 of 135 [11.1%] [difference, 24.0%; 95% CI, 16.5%-30.0%] and 29 of 156 [18.6%] [difference, 16.5%; 95% CI, 8.6%-23.3%]) (Table 2).

**Table 1.** Characteristics of the Total Study Population and Subgroups

Characteristic	Participants, No. (%)			
	All (n = 818)	Undocumented Latino immigrants (n = 136)	Latino LRCs (n = 160)	Non-Latino LRCs (n = 522)
Age, median (IQR), y	50 (37-62)	46 (35.7-55.3)	46 (31-59)	53 (40-65)
Gender				
Man	338 (41.4)	57 (41.9)	58 (36.3)	223 (42.9)
Woman	472 (57.8)	79 (58.1)	98 (61.3)	295 (56.7)
Other or prefer not to answer^a^	6 (0.7)	0	4 (2.5)	2 (0.4)
Primary language				
English	609 (74.4)	9 (6.6)	101 (63.1)	499 (95.6)
Spanish	185 (22.6)	126 (92.6)	57 (35.6)	2 (0.4)
Other^b^	24 (2.9)	1 (0.7)	2 (1.3)	21 (4.0)
Unstable housing				
Yes	766 (93.6)	122 (89.7)	149 (93.1)	495 (94.8)
No	39 (4.8)	9 (6.6)	6 (3.8)	24 (4.6)
Marginal	13 (1.6)	5 (3.7)	5 (3.1)	3 (0.6)
Insurance status^c^				
Private	270 (28.9)	5 (3.7)	26 (16.3)	239 (45.8)
Medicare	183 (19.6)	0	26 (16.3)	157 (30.1)
Medicaid	272 (29.1)	57 (41.9)	68 (42.5)	147 (28.2)
ObamaCare	6 (0.6)	0	6 (3.8)	0
Military or VA	3 (0.3)	0	0	3 (0.6)
Kaiser	1 (0.1)	0	0	1 (0.2)
City or county^d^	100 (10.7)	45 (33.1)	24 (15.0)	31 (5.9)
Other	17 (1.8)	0	7 (4.4)	10 (1.9)
Uninsured	69 (7.4)	35 (25.7)	13 (8.1)	21 (4.0)
Unsure	14 (1.5)	4 (2.9)	4 (2.5)	6 (1.1)
Have primary care				
Yes	691 (84.6)	96 (70.6)	126 (79.2)	469 (89.8)
No	121 (14.8)	39 (28.7)	32 (20.1)	50 (9.6)
Unsure	5 (0.6)	1 (0.7)	1 (0.6)	3 (0.6)
Long COVID				
Yes	220 (27.2)	42 (30.9)	33 (20.9)	145 (28.1)
No	564 (69.6)	94 (69.1)	112 (70.9)	358 (69.4)
Unsure	26 (3.2)	0	13 (8.2)	13 (2.5)

Abbreviations: LRCs, legal residents or citizens; VA, US Department of Veterans Affairs.

^a^ Includes nonbinary, transgender female, transgender male, or not listed.

^b^ Includes Cantonese or Mandarin, Tagalog, Arabic, Bengali, or other.

^c^ Participants could select more than 1 insurance.

^d^ City or county insurance are programs in certain cities (eg, Health SF, San Francisco) funded by the Department of Public Health that provide free or affordable health services to those who are ineligible for public insurance and earn below a certain income threshold.

**Table 2.** Understanding and Knowledge of Long COVID

Understanding and knowledge	Participants, No. (%)			
	All (n = 818)	Undocumented Latino immigrants (n = 135)	Latino LRCs (n = 156)	Non-Latino LRCs (n = 518)
Knowledge of long COVID				
Yes	303 (37.5)	25 (18.5)	47 (30.1)	231 (44.6)
No	489 (60.4)	109 (80.7)	105 (67.3)	275 (53.1)
Unsure	17 (2.1)	1 (0.7)	4 (2.6)	12 (2.3)
Understanding of long COVID				
Yes	226 (70.4)	15 (57.7)	29 (56.9)	182 (74.6)
No	51 (15.9)	9 (34.6)	11 (21.6)	31 (12.7)
Somewhat	44 (13.7)	2 (7.7)	11 (21.6)	31 (12.7)
Knowledge and understanding of long COVID^a^				
Yes	226 (27.9)	15 (11.1)	29 (18.6)	182 (35.1)
No	583 (72.1)	120 (88.9)	127 (81.4)	336 (64.9)

Abbreviation: LRCs, legal residents and citizens.

^a^ Participants who answered yes or unsure to having familiarity with long COVID and answered yes to having knowledge of what long COVID is were categorized as having knowledge and understanding of long COVID.

Prevalence of long COVID symptoms was 30.9% (n = 42) among undocumented Latino immigrants, 20.9% (n = 33) among Latino LRCs, and 28.1% (n = 145) among non-Latino LRCs. Rate of missing work or school due to long COVID symptoms was highest among undocumented Latino immigrants (20 of 35 [57.1%]) followed by non-Latino LRCs (56 of 121 [46.3%]) and Latino LRCs (10 of 28 [35.7%]). Rates of lack of care for long COVID symptoms were 60.0% (n = 24) among undocumented Latino immigrants, 63.6% (n = 21) among Latino LRCs, and 43.4% (n = 63) among non-Latino LRCs.

## Discussion

This study found that despite the high prevalence and impact of long COVID symptoms, undocumented Latino immigrants had limited knowledge and understanding of long COVID; 60.0% did not receive care for prior long COVID symptoms. Limitations include analysis of only undocumented immigrants identifying as Latino; thus, generalizability is limited to this group. Long COVID has a dynamic and evolving definition; we used the CDC-accepted definition at the time of writing.

Our findings provide insight into understanding of and access to care for long COVID among undocumented Latino immigrants, highlighting the need for education and follow-up care for this group. We recommend culturally relevant and translated resources and protocols in the ED to ensure access to diagnosis and follow-up care for long COVID. Future investigations prioritizing inclusion of undocumented people are needed to enhance applicability and generalizability of findings.

## Notes

Supplement 1. **eMethods.** Exclusions **eAppendix 1.** Survey Instrument – English **eAppendix 2.** Survey Instrument – Spanish

Supplement 2.
Data Sharing Statement
