# Supplementary material for: Long COVID Among Undocumented Latino Immigrant Populations in the Emergency Department
Source: JAMA Netw Open. 2024 Oct 11;7(10):e2438806. doi: 10.1001/jamanetworkopen.2024.38806 (PMC11470391; doi:10.1001/jamanetworkopen.2024.38806)
Supplement: Supplement 1. — eMethods. Exclusions eAppendix 1. Survey Instrument – English eAppendix 2. Survey Instrument – Spanish [file jamanetwopen-e2438806-s001.pdf]

Supplemental Online Content

Reyes KP, Rafique Z, Chinnock B, et al. Long COVID among undocumented Latino immigrant populations in the emergency department. 2024;7(10):e2438806.
doi:10.1001/jamanetworkopen.2024.38806

eMethods. Exclusions

eAppendix 1. Survey Instrument – English

eAppendix 2. Survey Instrument – Spanish

This supplemental material has been provided by the authors to give readers additional information about their work.

© 2024 Reyes KP et al. *JAMA Network Open*

eMethods. Exclusions

1. Age < 18 years
2. Presentation for intoxication or overdose
3. Inability to participate in a survey because of intoxication, altered mental status, or critical illness
4. Incarceration
5. Psychiatric hold
6. In respiratory or contact isolation
7. Patients with a new positive Covid test in the ED
8. Presenting to the ED with non-Covid related complaints such as but not limited to trauma (major or minor - lacerations, sprains, fractures, skin infections etc.), eye problems, cellulitis/abscess, arm or leg pain or swelling, wound check/wound infection/suture removal, post op problem, nosebleed, foreign object, dysuria, hematuria, melena, vaginal bleeding, pregnancy related issues.

© 2024 Reyes KP et al. *JAMA Network Open*

eAppendix 1. Survey Instrument – English

1. Subject ID ________________
2. Date of survey_____________
3. Have you ever had a positive test for Covid?
    a. Yes
    b. No
    c. Possibly
    d. I don't remember
4. **If yes to 3**, how many times? _______
5. **If yes to 3**, how long ago did you have it the last time?
    a. Less than a month
    b. 1-3 months
    c. 3-6 months
    d. 6 months to a year
    e. 1-2 years ago
    f. > 2 years ago
6. **If yes to 3**, where did you have the COVID test the last time?
    a. Home test
    b. Test at a clinic
    c. Test at an ER
    d. Test at a testing site
    e. Other__________________
7. Did you ever have to come to an emergency department when you were sick with Covid?
    a. Yes
    b. No
    c. I don't remember
8. Did you ever have to stay in the hospital overnight when you were sick with Covid?
    a. Yes
    b. No
    c. I don't remember
9. Did you have any symptoms or problems that lasted longer than a month after you were sick with Covid?
    a. Yes
    b. No
    c. Possibly lasted longer than a month, but I am not sure exactly
    d. I don't remember
10. **If yes or possibly to question 9**, what were these symptoms? (Select all that apply)
    - ☐ Fatigue/Tiredness/Malaise
    - ☐ Cough
    - ☐ Chest Pain
    - ☐ Shortness of breath
    - ☐ Fever
    - ☐ Difficulty concentrating or thinking
    - ☐ Headache
    - ☐ Abdominal pain

© 2024 Reyes KP et al. *JAMA Network Open*

- ☐ Diarrhea
- ☐ Depression/Anxiety
- ☐ Dizziness/Lightheadedness
- ☐ Sleep disturbances
- ☐ Changes in menstrual cycle
- ☐ Muscle aches/Joint Pains
- ☐ Loss of Taste
- ☐ Loss of Smell
- ☐ Other

11. **(CRC will ask this question for each individual symptom checked in question 10)** How long did each of these symptoms last after you had Covid?

	I still have this symptom now	1-3 months	3-6 months	6 months to a year	-2 Years	>2 years
Fatigue/Tiredness/Malaise	○	○	○	○	○	○
Cough	○	○	○	○	○	○
Chest Pain	○	○	○	○	○	○
Shortness of breath	○	○	○	○	○	○
Fever	○	○	○	○	○	○
Difficulty concentrating or thinking	○	○	○	○	○	○
Headache	○	○	○	○	○	○
Abdominal pain	○	○	○	○	○	○
Diarrhea	○	○	○	○	○	○
Depression/Anxiety	○	○	○	○	○	○
Dizziness/Lightheadedness	○	○	○	○	○	○
Sleep disturbances	○	○	○	○	○	○
Changes in menstrual cycle	○	○	○	○	○	○
Muscle aches/Joint Pains	○	○	○	○	○	○
Loss of Taste	○	○	○	○	○	○

© 2024 Reyes KP et al. *JAMA Network Open*

Loss of Smell	○	○	○	○	○	○
Other	○	○	○	○	○	○

12. **(If yes or possibly to question 9)**, Are any of these symptoms that lasted longer than a month a reason you are here in the emergency department today?
    a. Yes
        i. Which ones are a reason you are here in the emergency department?
    - ☐ Fatigue/Tiredness/Malaise
    - ☐ Cough
    - ☐ Chest Pain
    - ☐ Shortness of breath
    - ☐ Fever
    - ☐ Difficulty concentrating or thinking
    - ☐ Headache
    - ☐ Abdominal pain
    - ☐ Diarrhea
    - ☐ Depression/Anxiety
    - ☐ Dizziness/Lightheadedness
    - ☐ Sleep disturbances
    - ☐ Changes in menstrual cycle
    - ☐ Muscle aches/Joint Pains
    - ☐ Loss of Taste
    - ☐ Loss of Smell
    - ☐ Other

    b. No
    c. Unsure (possibly, maybe, they might be a reason)
        i. Which symptoms might be a reason you are here in the emergency department?
    - ☐ Fatigue/Tiredness/Malaise
    - ☐ Cough
    - ☐ Chest Pain
    - ☐ Shortness of breath
    - ☐ Fever
    - ☐ Difficulty concentrating or thinking
    - ☐ Headache
    - ☐ Abdominal pain
    - ☐ Diarrhea
    - ☐ Depression/Anxiety
    - ☐ Dizziness/Lightheadedness
    - ☐ Sleep disturbances
    - ☐ Changes in menstrual cycle
    - ☐ Muscle aches/Joint Pains
    - ☐ Loss of Taste
    - ☐ Loss of Smell

© 2024 Reyes KP et al. *JAMA Network Open*

- ☐ Other

13. **If yes or possibly to question 9,** did you have to miss work or school because of these prolonged symptoms?
    a. Yes, I had to miss work
    b. Yes, I had to miss school.
    c. Yes, I had to miss work and school.
    d. No, I didn't have to miss work
    e. No, I didn't have to miss school.
    f. Unsure
    g. I was (am) not working at the time.
    h. I was (am) not going to school at the time.

14. How long did you have to miss work or school? __________
15. Have you ever had a Covid vaccine?
    a. Yes
    b. No
    c. Unsure
16. **If yes,** how many have you had?
    a. 1
    b. 2
    c. 3
    d. 4
    e. 5 or more
17. **If yes,** when was your last Covid vaccine?
    a. 0-3 months ago
    b. 3-6 months ago
    c. 6 months to a year ago
    d. 1-2 years ago
    e. > 2 years ago
    f. Unsure

18. Have you ever heard of "*Long Covid*"?
    a. Yes
    b. No
    c. Unsure

19. **If yes to 18**, do you think you had or currently have Long Covid?
    a. Yes, I had it but it's gone now.
    b. Yes, I have it now.
    c. No
    d. Unsure
20. **If yes or unsure to 19**, which symptoms of Long Covid do you think you have had?
    - ☐ Fatigue/Tiredness/Malaise
    - ☐ Cough
    - ☐ Chest Pain
    - ☐ Shortness of breath

© 2024 Reyes KP et al. *JAMA Network Open*

- ☐ Fever
- ☐ Difficulty concentrating or thinking
- ☐ Headache
- ☐ Abdominal pain
- ☐ Diarrhea
- ☐ Depression/Anxiety
- ☐ Dizziness/Lightheadedness
- ☐ Sleep disturbances
- ☐ Changes in menstrual cycle
- ☐ Muscle aches/Joint Pains
- ☐ Loss of Taste
- ☐ Loss of Smell
- ☐ Other

21. **If yes to 19,** have you ever seen or doctor, health care provider, clinic or ER for Long Covid symptoms?
    a. Yes
        i. Where?
            1. A clinic
            2. An ER
            3. Other_________________
    b. No
    c. Unsure

---

22. How old are you? ___________
23. What is your gender?
    a. Male
    b. Female
    c. Transgender male
    d. Transgender female
    e. Non-binary/gender non-conforming
    f. Not listed ________________
    g. Prefer not to answer
24. Are you of Hispanic or Latino Origin?
    a. Yes
    b. No
    c. Prefer not to answer
25. Which best describes your race? Check all that apply.
    a. American Indian/Alaska Native
    b. Asian [SEE BRANCHING LOGIC]
        1, Chinese
        2, Filipino
        3, Asian Indian
        4, Vietnamese
        5, Korean

6, Japanese
7, Other Asian
c. Black or African American
d. Native Hawaiian/Other Pacific Islander [SEE BRANCHING LOGIC]
1, Native Hawaiian
2, Samoan
3, Chamorro
4, Other Pacific Islander (e.g., Tongan, Fijian, Marshallese)
e. White
f. Other race (specify): ___________________

26. What is your living situation today?
    a. I have a steady place to live (home, apartment or other)
    b. I have a place to live today, but I am worried about losing it in the future
    c. I do not have a steady place to live (I am temporarily staying with others, in a hotel, in a shelter, living outside on the street, on a beach, in a car, abandoned building, bus or train station, or in a park).
27. Do you have any kind of health care coverage, including health insurance, prepaid plans such as HMOs, or government plans such as Medicaid, Medicare, or Indian Health Service? CHECK ALL THAT APPLY
    a. I do not have health insurance
    b. Medicare
    c. Medicaid/State Insurance
    d. Obamacare (Affordable Care Act)
    e. Military (VA)
    f. Private/Commercial/Employer-based/Self-insured
    g. Kaiser
    h. Indian Health Service
    i. Healthy San Francisco (or other similar)
    j. Other insurance
28. Do you have a regular clinic or doctor for medical care?
    a. Yes
    b. No
    c. Unsure
29. **<u>If NO to regular doctor or clinic,</u>** when was the last time you saw *<u>any</u>* doctor or had health care in the U.S. (besides today)?
    a. < 6 months
    b. < 6 months to a year
    c. 1 year to 5 years
    d. > 5 years
    e. I have never seen a doctor in the U.S.

30. **<u>If NO to regular doctor or clinic,</u>** where do you usually go when you are sick or need medical advice?
    a. An emergency department
    b. A clinic
    c. Urgent care center
    d. Other____________

© 2024 Reyes KP et al. *JAMA Network Open*

e. I don't ever go anywhere
f. I have never been sick
g. Unsure

31. What is your primary language?
a. English
b. Spanish
c. Cantonese/Mandarin
d. Tagalog
e. Arabic
f. Bengali
g. Other ______________

32. Are you a US citizen?
a. Yes
b. No
c. Declined to answer
d. Unsure
e. Resident

© 2024 Reyes KP et al. *JAMA Network Open*

eAppendix 2. Survey Instrument – Spanish

1. ID Del Participante ________________
2. Fecha de la encuesta______________
3. ¿Alguna vez ha tenido una prueba positiva de COVID?
    a. Si
    b. No
    c. Posiblemente
    d. No me acuerdo
4. **Si respondió "si"**, ¿Cuantas veces? _______
5. **Si respondió "si"**, ¿Por cuánto tiempo lo tuvo la última ves?
    a. Menos de 1 mes
    b. 1-3 meses
    c. 3-6 meses
    d. 6 meses a 1 año
    e. Hace 1-2 años
    f. > 2 años
6. **Si respondió "si: a la pregunta #3,** ¿dónde se hizo la prueba de COVID la última vez?
    a. Prueba en casa
    b. Prueba en la clínica
    c. Prueba en la sala de emergencias
    d. En un sitio para pruebas
    e. Otro_______
7. ¿Alguna vez tuviste que acudir a un departamento de emergencia cuando estaba enfermo con COVID?
    a. Si
    b. No
    c. No me acuerdo
8. ¿Alguna vez fuiste hospitalizado cuando estaba enfermo con COVID?
    a. Si
    b. No
    c. No me acuerdo
9. ¿Tuvo algún síntoma o problema que duró más de un mes después de enfermarse de COVID?
    a. Si
    b. No
    c. Posiblemente duro más que un mes, pero no estoy seguro exactamente
    d. No me acuerdo
10. **Si respondió "si" o "posiblemente" a la pregunta 9**, ¿Que síntomas fueron? (Seleccione todos los que aplican)
    - Fatiga/cansancio/malestar general
    - Tos
    - Dolor de pecho
    - Dificultad para respirar
    - Fiebre
    - Dificultad para concentrarse o pensar

© 2024 Reyes KP et al. *JAMA Network Open*

- Dolor de cabeza
- Dolor estomago
- Diarrea
- Depresión/Ansiedad
- Mareos/Aturdimiento
- Trastornos del sueño
- Cambios en el ciclo menstrual
- Dolores musculares/dolores articulaciones
- Perdida de oler
- Perdida de sabor
- Otro

11. **(CRC hará esta pregunta para cada síntoma individual marcado en la pregunta 10)** ¿Cuánto duro cada síntoma después de que tuviste COVID?

	Todavía tengo este síntoma	1-3 meses	3-6 meses	6 meses a un año	1-2 año	>2 año
Fatiga/cansancio/malestar general	○	○	○	○	○	○
Tos	○	○	○	○	○	○
Dolor de pecho	○	○	○	○	○	○
Dificultad para respirar	○	○	○	○	○	○
Fiebre	○	○	○	○	○	○
Dificultad para concertarse o pensar	○	○	○	○	○	○
Dolor de cabeza	○	○	○	○	○	○
Dolor estomago	○	○	○	○	○	○
Diarrea	○	○	○	○	○	○
Depresión/Ansiedad	○	○	○	○	○	○
Mareos/Aturdimiento	○	○	○	○	○	○
Trastornos del sueño	○	○	○	○	○	○
Cambios en el ciclo menstrual	○	○	○	○	○	○

© 2024 Reyes KP et al. *JAMA Network Open*

Dolores musculares/ dolores articulaciones	○	○	○	○	○	○
Perdida de oler	○	○	○	○	○	○
Perdida de sabor	○	○	○	○	○	○
Otro	○	○	○	○	○	○

12. **(Si respondió "si" o "posiblemente..." a la pregunta 9)**, ¿Alguno de estos síntomas que duraron más de un mes es una razón por la cual usted está en el departamento de emergencias hoy?
   a. Si
      i. ¿Cuáles síntomas son la razón por la cual usted está aquí en el departamento de emergencias?
         - Fatiga/cansancio/malestar general
         - Tos
         - Dolor de pecho
         - Dificultad para respirar
         - Fiebre
         - Dificultad para concentrarse o pensar
         - Dolor de cabeza
         - Dolor estomago
         - Diarrea
         - Depresión/Ansiedad
         - Mareos/Aturdimiento
         - Trastornos del sueño
         - Cambios en el ciclo menstrual
         - Dolores musculares/dolores articulaciones
         - Perdida de oler
         - Perdida de sabor
         - Otro
   b. No
   c. No estoy seguro (posiblemente, quizás, puede que sean una razón)
      i. ¿Cuáles síntomas son la razón por la cual usted está aquí en el departamento de emergencias?
         - Fatiga/cansancio/malestar general
         - Tos
         - Dolor de pecho
         - Dificultad para respirar
         - Fiebre
         - Dificultad para concentrarse o pensar
         - Dolor de cabeza
         - Dolor estomago
         - Diarrea
         - Depresión/Ansiedad

© 2024 Reyes KP et al. *JAMA Network Open*

- Mareos/Aturdimiento
- Trastornos del sueño
- Cambios en el ciclo menstrual
- Dolores musculares/dolores articulaciones
- Perdida de oler
- Perdida de sabor
- Otro

13. **Si respondió "sí" o "posiblemente" a la pregunta 9,** ¿tuvo que faltar al trabajo o a la escuela debido a estos síntomas prolongados?
    a. Sí, tuve que faltar al trabajo.
    b. Sí, tuve que faltar a la escuela.
    c. Sí, tuve que faltar al trabajo ya la escuela.
    d. No, no tuve que faltar al trabajo
    e. No, no tenía que faltar a la escuela.
    f. No estoy seguro / No estoy seguro
    g. No estaba (no estoy) trabajando en ese momento.
    h. No estaba (estoy) yendo a la escuela en ese momento.
14. **Si respondió "si"**, ¿cuánto tiempo falto al trabajo o a la escuela? ______
15. ¿Ha vacunado contra el COVID?
    a. Si
    b. No
    c. No estoy seguro
16. **Si respondió "si"**, ¿Cuantas vacunas a recibido en total?
    a. 1
    b. 2
    c. 3
    d. 4
    e. Mas de 5
17. **Si respondió "si",** ¿Cuándo fue la última vez que se vacuno conta el COVID?
    a. Hace 0-3 meses
    b. Hace 3-6 meses
    c. Hace 6 meses o 1 año
    d. Hace 1-2 años
    e. Hace más de 2 años
18. ¿Ha escuchado hablar de COVID por largo plazo, *"long covid"?*
    f. Si
    g. No
    h. No estoy seguro
19. **Si respondió "si" a la pregunta 18**, ¿Piensa que tiene o a tenido COVID por largo tiempo?
    a. Si lo tuve, pero ya no
    b. Si, lo tengo ahora
    c. No
    d. No estoy seguro
20. **Si respondió "si" o "no estoy seguro" a la pregunta 19**, ¿Que síntomas de COVID a largo plazo piensa que ha tenido?
    - Fatiga/cansancio/malestar general
    - Tos

© 2024 Reyes KP et al. *JAMA Network Open*

- Dolor de pecho
- Dificultad para respirar
- Fiebre
- Dificultad para concentrarse o pensar
- Dolor de cabeza
- Dolor estomago
- Diarrea
- Depresión/Ansiedad
- Mareos/Aturdimiento
- Trastornos del sueño
- Cambios en el ciclo menstrual
- Dolores musculares/dolores articulaciones
- Perdida de oler
- Perdida de sabor
- Otro

21. **Si respondió "si" a la pregunta 19**, ¿Ha visitado a un doctor, proveedor de atención médica, clínica o sala de emergencias por sus síntomas de COVID por largo tiempo?
    a. Si
        i. ¿Donde?
            1. Una clínica
            2. La sala de emergencias
            3. Otro________
    b. No
    c. No estoy seguro

---

22. ¿Cuántos años tiene? ___________
23. ¿Cuál es su género?
    a. Hombre
    b. Mujer
    c. Hombre Transgénero
    d. Mujer Transgénero
    e. No-binario/genero no-conforme
    f. No esta enlistado ________________
    g. Prefiero no responder
24. ¿Es de origen Latina o Hispana?
    a. Si
    b. No
    c. Prefiero no contestar
25. ¿Cuál describe su raza mejor? (Seleccione todo lo que aplique)
    a. Indio Americano/Nativo de Alaska
    b. Asiático [Vea la lógica de ramificación]
        1, chino
        2, filipino
        3, indio asiático
        4, vietnamita
        5, coreano

© 2024 Reyes KP et al. *JAMA Network Open*

6, japonés
7, otro asiático (Pakistaní, Camboyano, Hmong)
c. Negro o Africano Americano
d. Nativo de Hawái/Otro islandés del Pacifico [Vea la lógica de ramificación]
1, nativo hawaiano
2, samoano
3, chamorro
4, otro isleño del Pacífico (p. ej., tongano, fiyiano, marshalés)
e. Blanco
f. Otra raza (especificar): ___________________

26. ¿Cuál es su situación de vivienda hoy?
a. Yo tengo un lugar estable para vivir (Casa, apartamento o otro)
b. Yo tengo un lugar para vivir hoy, pero tengo miedo de perderlo en el futuro
c. Yo no tengo un lugar estable donde vivir (Temporalmente me estoy quedado con otros, en un hotel, en un albergue, viviendo afuera en la calle, en la playa, en un caro, edificio abandonado, autobús o estación de tren, o en el parque).

27. ¿Tiene algún tipo de seguro de salud, incluyendo seguranza médica, planes prepagados como HMOs, o planes del gobierno como Medicaid, Medicare, o Servicio de salud indio? [SELECCIONE TODO LO QUE APLIQUE]
a. No tengo seguranza medica
b. Medicare
c. Medicaid/Seguranza estatal
d. Obamacare (Affordable Care Act)
e. Militar (VA)
f. Privada/Comercial/Basado en su empleador/Auto asegurado
g. Kaiser
h. Servicio de salud indio
i. Seguro de ciudad/condado (p.ej. Healthy San Francisco o otro similar)
j. Otra seguranza

28. ¿Tiene una clínica primaria o medico primario donde recibe su cuidado médico?
a. Si
b. No
c. No estoy seguro

29. **Si la respuesta es NO,** ¿Cuándo fue la última vez que vio a un médico o recibió atención médica en los EE. UU. (Además de hoy)?
a. < 6 meses
b. < 6 meses a 1 año
c. 1 año a 5 años
d. > 5 años
e. Nunca he visto a un médico en los EE. UU.

30. **Si la respuesta es NO al médico de cabecera,** ¿A dónde va cuando está enfermo o necesita atención médica?
a. El Departamento de Emergencias
b. Una clínica
c. Centro de atención de urgencias
d. Otro__________

© 2024 Reyes KP et al. *JAMA Network Open*

e. Nunca voy a ninguna parte
f. Nunca he estado enfermo
g. No estoy seguro

31. ¿Cuál es su lenguaje principal?
    a. Ingles
    b. Español
    c. Cantonés/mandarín
    d. Tagalo
    e. Árabe
    f. Bengalí
    g. Otro:
32. Eres ciudadano de los Estados Unidos?
    a. Si
    b. No
    c. Se nego a responder
    d. No estoy seguro
    e. Residente

© 2024 Reyes KP et al. *JAMA Network Open*
