# Supplementary material for: Long COVID Among Undocumented Latino Immigrant Populations in the Emergency Department
Source: JAMA Netw Open. 2024 Oct 11;7(10):e2438806. doi: 10.1001/jamanetworkopen.2024.38806 (PMC11470391; doi:10.1001/jamanetworkopen.2024.38806)
Supplement: Supplement 2. — Data Sharing Statement [file jamanetwopen-e2438806-s002.pdf]

Data Sharing Statement

Reyes. Long COVID Among Undocumented Latino Immigrant Populations in the Emergency Department. *JAMA Netw Open*. Published October 11, 2024.
doi:10.1001/jamanetworkopen.2024.38806

Data
Data available: Yes
Data types: Deidentified participant data
How to access data: For data reach out to karen.reyes@ucsf.edu or robert.rodriguez@ucsf.edu
When available: With publication

Supporting Documents
Document types: None

Additional Information
Who can access the data: Researchers whose proposed use of the data has been approved.
Types of analyses: Any pre-approved purpose.
Mechanisms of data availability: After approval of proposal.
